# Dystrophin S3059 phosphorylation partially attenuates denervation atrophy in mouse tibialis anterior muscles

**DOI:** 10.14814/phy2.16145

**Published:** 2024-07-12

**Authors:** Kristy Swiderski, Timur Naim, Jennifer Trieu, Annabel Chee, Marco J. Herold, Andrew J. Kueh, Craig A. Goodman, Paul Gregorevic, Gordon S. Lynch

**Affiliations:** ^1^ Department of Anatomy and Physiology, Centre for Muscle Research The University of Melbourne Melbourne Victoria Australia; ^2^ The Walter and Eliza Hall Institute of Medical Research Parkville Victoria Australia; ^3^ Department of Medical Biology The University of Melbourne Melbourne Victoria Australia; ^4^ Olivia Newton‐John Cancer Research Institute Heidelberg Victoria Australia; ^5^ School of Cancer Medicine La Trobe University Heidelberg Victoria Australia

**Keywords:** denervation, dystrophin, muscle atrophy, phosphorylation, S3059

## Abstract

The dystrophin protein has well‐characterized roles in force transmission and maintaining membrane integrity during muscle contraction. Studies have reported decreased expression of dystrophin in atrophying muscles during wasting conditions, and that restoration of dystrophin can attenuate atrophy, suggesting a role in maintaining muscle mass. Phosphorylation of S3059 within the cysteine‐rich region of dystrophin enhances binding between dystrophin and β‐dystroglycan, and mimicking phosphorylation at this site by site‐directed mutagenesis attenuates myotube atrophy in vitro. To determine whether dystrophin phosphorylation can attenuate muscle wasting in vivo, CRISPR‐Cas9 was used to generate mice with whole body mutations of S3059 to either alanine (DmdS3059A) or glutamate (DmdS3059E), to mimic a loss of, or constitutive phosphorylation of S3059, on all endogenous dystrophin isoforms, respectively. Sciatic nerve transection was performed on these mice to determine whether phosphorylation of dystrophin S3059 could attenuate denervation atrophy. At 14 days post denervation, atrophy of tibialis anterior (TA) but not gastrocnemius or soleus muscles, was partially attenuated in DmdS3059E mice relative to WT mice. Attenuation of atrophy was associated with increased expression of β‐dystroglycan in TA muscles of DmdS3059E mice. Dystrophin S3059 phosphorylation can partially attenuate denervation‐induced atrophy, but may have more significant impact in less severe modes of muscle wasting.

## Graphical abstract

Mice expressing systemic phospho‐null (DmdS3059A) or phosphomimetic (DmdS3059E) mutation of the dystrophin protein were generated using CRISPR/Cas9 technology and subjected to sciatic nerve transection. Denervation‐induced atrophy was partially attenuated in the tibialis anterior muscles of mice expressing the DmdS3059E mutation. Attenuation of atrophy was associated with increased expression of β‐dystroglycan. Dystrophin S3059 phosphorylation can partially attenuate denervation‐induced atrophy, but may have more significant impact in less severe modes of muscle wasting. Created with BioRender.com.

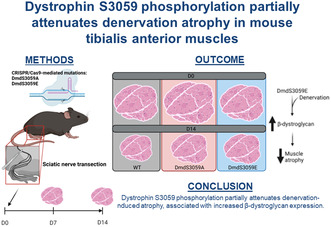

## 1 INTRODUCTION

The regulation of skeletal muscle mass is a complex process modulated by signaling pathways controlling protein synthesis and protein degradation (Lloyd, 2013). Understanding these pathways during muscle wasting conditions is essential for developing interventions to attenuate muscle atrophy. The dystrophin glycoprotein complex (DGC) is a multimeric protein complex linking the extracellular matrix to the actin cytoskeleton, involved in force transmission across the muscle fiber membrane and potentially as a regulator of muscle mass (Ervasti & Campbell, 1993). Decreased expression of DGC proteins, especially dystrophin, have been reported in conditions associated with muscle atrophy, including sarcopenia, tenotomy, and cancer (Acharyya et al., 2005; Campelj et al., 2020; Chockalingam et al., 2002; Hord et al., 2016; Kosek & Bamman, 2008). Tumor‐associated muscle wasting is exacerbated in mice lacking dystrophin, but restoration of dystrophin expression can attenuate this muscle wasting (Acharyya et al., 2005). Increasing DGC protein expression and/or localization to confer stability at the membrane may have therapeutic implications for muscle wasting disorders.

Phosphorylation of the DGC proteins, including β‐dystroglycan (Abdullah et al., 2020; Ilsley et al., 2001; James et al., 2000; Miller et al., 2012; Sotgia et al., 2001), utrophin (Ramirez et al., 2023), α1‐syntrophin (Madhavan & Jarrett, 1999; Zhou et al., 2006), and dystrophin (Calderilla‐Barbosa et al., 2006; Fujimoto et al., 2020; Madhavan & Jarrett, 1994, 1999; Michalak et al., 1996; Milner et al., 1993; Senter et al., 1995; Shemanko et al., 1995; Swiderski et al., 2014, 2021; Wagner & Huganir, 1994), modulate the stability of the DGC and may influence skeletal muscle mass. We showed that phosphorylation of S3059 on the dystrophin protein increased the binding affinity for dystrophin to β‐dystroglycan (Swiderski et al., 2014). Furthermore, mimicking this phosphorylation event by mutating S3059 to glutamate (S3059E) attenuated muscle wasting in vitro after exposure to C‐26 cancer cells or interleukin (IL)‐6 (Swiderski et al., 2021). Whether modulating DGC protein expression and/or stability can attenuate muscle atrophy in models of wasting in vivo, and whether this is influenced by the factors initiating muscle atrophy, remains unknown.

Denervation with sciatic nerve transection leads to rapid and severe atrophy and dysfunction of denervated skeletal muscles, associated with increased protein degradation and, somewhat paradoxically, increased protein synthesis (Hornberger et al., 2001; Machida et al., 2012). While activation of proteasomal and autophagic signaling has been well characterized in experimental models of denervation, with reports of increased mTORC1 signaling (Bentzinger et al., 2013; Castets et al., 2019; Goodman et al., 2017; Quy et al., 2013; Tang et al., 2014; You et al., 2021), other conflicting reports suggest this reflects an attempt at a compensatory hypertrophic response to counter the enhanced atrophy signaling (Quy et al., 2013; Tang et al., 2014). Surprisingly, while studies have examined the impact of denervation in dystrophin‐deficient skeletal muscles (Anderson, 1991; Jasmin et al., 1995; Mechalchuk & Bressler, 1992; Mitsui et al., 1996; Suh et al., 1994; Takemitsu et al., 1991; Xu & Salpeter, 1997), there is no evidence that atrophy is more severe after denervation in muscles deficient in dystrophin or lacking DGC integrity. In rats, sciatic nerve transection decreased dystrophin protein expression in homogenates from soleus muscles over a period of 15 days, yet in gastrocnemius muscles, dystrophin protein expression was increased, suggesting differential regulation of the DGC in response to denervation in faster versus slower skeletal muscles (Boudriau et al., 1996). Furthermore, in dystrophin deficient *mdx* mice, denervation upregulated DGC protein expression in atrophying muscle fibers, particularly β‐dystroglycan, suggesting that even in the absence of dystrophin, denervation alters DGC protein expression and localization (Mitsui et al., 1996). Together, these studies suggest the DGC may be involved in the regulation of muscle mass after denervation, but whether increasing DGC stability, through modulation of the interaction between dystrophin and β‐dystroglycan via phosphorylation, affects denervation‐induced atrophy, is unknown.

To interrogate this question, mice were generated with CRISPR‐induced mutations in the endogenous dystrophin protein resulting in body‐wide expression of dystrophin protein isoforms with either an alanine (S3059A) or glutamate (S3059E) substitution at S3059. This facilitated investigation of whether loss of phosphorylation (S3059A) or mimicking phosphorylation (S3059E), respectively, at this site, altered the muscle atrophy response to unilateral hindlimb denervation.

## 2 METHODS

### 2.1 Animals

All experiments were approved by the Animal Ethics Committee of The University of Melbourne and conducted in accordance with the Australian code for the care and use of animals for scientific purposes as stipulated by the National Health and Medical Research Council (Australia). DmdS3059A and DmdS3059E mutant mice were created by Prof. Marco Herold and Dr. Andrew Kueh at the MAGEC facility (Walter and Eliza Hall Institute, Melbourne, Australia). To generate mice, 20 ng/μL of Cas9 mRNA, 10 ng/μL of sgRNA (GGGCACTTTGTTTGGTGAGA) and 40 ng/μL of oligo donor (DmdS3059A: tttcttttctttttcctttttttctttttgcagCTTCAGTTCAGGGTCCCTGGGAGAGAGCtATCGCCCCAAACAAAGTGCCCTACTATATCAAgtaagtcaaaagcatttatgtacctgatctgtat; DmdS3059E: tttcttttctttttcctttttttctttttgcagCTTCAGTTCAGGGTCCCTGGGAGAGAGCtATCGAGCCAAACAAAGTGCCCTACTATATCAAgtaagtcaaaagcatttatgtacctgatctgtat) were injected into the cytoplasm of fertilized one‐cell stage embryos derived from WT C57BL/6 breeders. Viable founder mice were identified by next‐generation sequencing. Targeted animals were backcrossed onto C57BL/6 animals for two generations to eliminate potential sgRNA off‐target hits. Mice homozygous for either the S‐A or S‐E mutation were subsequently bred to maintain homozygous colonies. Male C57BL/6 WT mice were obtained from the Animal Resources Centre (Canning Vale, Western Australia) and DmdS3059A and DmdS3059E mice were bred in the Biological Research Facility at The University of Melbourne. All mice were housed in the Biological Research Facility under a 12:12‐h light–dark cycle, with water and standard laboratory chow (Barastoc Irradiated WEHI mice cubes; #8720610; Ridley Corp. Ltd., Melbourne, Australia) available ad libitum.

### 2.2 Denervation surgery

Twelve‐week‐old male C57BL/6 WT (*n* = 18), C57BL/6 DmdS3059A (*n* = 19), and C57BL/6 DmdS3059E (*n* = 22) were allocated into two groups: (i) sham control (*n* = 5–6); or (ii) denervated (*n* = 12–16). On Day 0, mice received sham surgery or denervation of the right hindlimb, in which ~2 mm portion of the sciatic nerve was excised at the mid‐thigh region. All animals received pre‐emptive analgesia via buprenorphine (0.05 mg/kg, *s.c*., 30 G needle). Thirty minutes later, the mice were anesthetized with isoflurane (3%–4% at 0.5 L/min for induction in chamber; maintained through a nose cone connected to oxygen‐isoflurane, 2%–3% at 0.5 L/min). Once non‐responsive to tactile stimuli (tail/toe pinch), fur around the right hindlimb was removed using clippers and the skin cleaned according to aseptic surgical techniques (chlorhexidine/hibitane application followed by ethanol). A small incision (2–8 mm) was made over the area below and parallel to the quadriceps muscles. The sciatic nerve was identified and the hindlimb muscles surgically denervated via transection of the sciatic nerve. The incision was closed with sterilized, surgical Michel clips (2–3 clips). On Day 7 (*n* = 6; denervated only) or Day 14 (*n* = 6–10; sham and denervated) post‐surgery, mice were anesthetized with sodium pentobarbital (60 mg/kg, *i.p*., ~200 μL volume, 30G needle) and the muscles of both denervated and non‐denervated hindlimbs excised carefully. Mice were killed via cardiac excision while deeply anesthetized.

### 2.3 Immunohistochemistry

After excision, the right TA muscle was bisected axially at the mid‐belly. One‐half of the right TA muscle was embedded in optimal cutting temperature (OCT) medium to prepare frozen sections. Serial sections (8 μm) were cut transversely through the TA muscle using a refrigerated (−20°C) cryostat (Minux FS800; RWD life science, Sugar Land, TX). For assessment of muscle fiber size, type and oxidative capacity, sections were immunostained as described previously (Murphy et al., 2019). Sections, frozen within 2 h after sectioning, were incubated in 300 mM NaH_2_PO_4_, 174 mM Na_2_HPO_4_ pH 7.6 containing 27 mg/mL sodium succinate (Sigma‐Aldrich, St. Louis, MO, USA) and 1 mg/mL nitrotetrazolium blue chloride (NBT; Sigma‐Aldrich) for 10 min at 37°C, rinsed 3× dH_2_O, dehydrated through 30%, 60%, and 90% acetone, and air dried for 15 min. Sections were then rinsed with 0.1% PBS + 0.1% Tween‐20 (PBST) for 10 min and incubated in primary antibodies including rabbit‐α‐laminin IgG (RRID: AB_477163; L9393; Sigma‐Aldrich), and mouse‐α‐MHC IIa IgG1 (SC‐71; RRID: AB_2147165) and mouse‐α‐MHC IIb IgM (BF‐F3; RRID: AB_2266724; both developed by S. Schiaffino, University of Padova (Padua, Italy), obtained from the Developmental Studies Hybridoma Bank), diluted at 1:25, 1:25, and 1:10, respectively, in 5% normal goat serum/0.05% PBST for 2 h at RT. Sections were washed 3× 5 min in PBS and incubated in secondary antibodies including AF647‐conjugated goat‐α‐mouse IgG1 (RRID: AB_2535809; A21240; Thermo Fisher Scientific, Waltham, MA, USA), AF488‐conjugated goat‐α‐mouse IgM (RRID: AB_2535711; A21042; Thermo Fisher Scientific), and AF555‐conjugated goat‐α‐rabbit IgG (RRID: AB_2535849; A21428; Thermo Fisher Scientific) diluted at 1:100, 1:250, and 1:250, respectively, in 5% normal goat serum/0.05% PBST for 1 h at RT. Sections were washed 3× 5 min in PBS, and mounted onto coverslips with Mowiol. Digital images of 4–5 non‐overlapping regions of stained sections were obtained using an upright microscope with camera (Axio Imager, Carl Zeiss, Wrek, Göttingen, Germany), controlled by ZEN software (ZEN 3.2 blue edition, Carl Zeiss). For quantification using FIJI/ImageJ software (Fiji v2.5.0, Fiji (imagej.net)), muscle fibers were outlined by thresholding of laminin‐stained images which were applied to the fiber type and SDH stained images to enable quantification of signal for individual muscle fibers. These analyses allowed for determination of the type of muscle fiber, based on antibody signal, and the minimum Feret's diameter of each muscle fiber.

### 2.4 qPCR

The remaining one‐half of the right TA muscle was snap frozen for gene expression analyses. Total RNA was extracted using TRIzol/chloroform followed by the RNeasy Mini Kit (#74016, Qiagen), as per manufacturer's instructions. The concentration and quality of RNA samples was determined using a Nanodrop 2000 spectrophotometer (Thermo Fisher Scientific). cDNA was generated from 160 ng RNA (with a 260/280 ratio between 1.8 and 2.1) using iScript Reverse Transcription Supermix (#1708841, Bio‐Rad Laboratories, Hercules, CA, USA), according to manufacturer's directions. Real‐time RT‐PCR was performed as described previously (Swiderski et al., 2016) using the following forward and reverse primer sequences: *MuRF1*—F 5′‐AGGTGTCAGCGAAAAGCAGT‐3′, R 5′‐CCTCCTTTGTCCTCTTGCTG‐3′, *Atrogin1*—F 5′‐GTTTTCAGCAGGCCAAGAAG‐3′, R 5′‐TTGCCAGAGAACACGCTATG‐3′, *LC3B*—F 5′‐CGGCTTCCTGTACATGGTTT‐3′, R 5′‐ATGTGGGTGCCTACGTTCTC‐3′. Gene expression was quantified using a cycle threshold (CT) method. Relative gene expression was calculated using the expression 2^−ΔCT^, and normalized to total cDNA content which was determined using the Quant‐iT™ OliGreen™ ssDNA assay kit and Quant‐iT OliGreen ssDNA reagent (#O11492, Thermo Fisher Scientific). Data are presented relative to WT for all timepoints.

### 2.5 Western immunoblotting

For analysis of muscle protein expression, the remaining right TA muscle was extracted from OCT and homogenized in ice‐cold buffer 10 mM Tris–HCl (pH 7.4), 100 mM NaCl, 1 mM EDTA, 1 mM EGTA, 1 mM NaF, 1% Triton, 10% glycerol, 0.1% SDS, 20 mM Na_4_P_2_O_7_, 0.5 mM Na_3_VO_4_, 0.5% sodium deoxycholate, 0.1 mM PMSF, phosphatase inhibitors (all from Sigma‐Aldrich) and protease inhibitor cocktail (#P2714, Sigma‐Aldrich) in a TissueLyser II (Qiagen, MD, USA) homogenizer using 5 mm stainless steel beads (Qiagen) for 2× 30 s pulses at 30 Hz. For analysis of protein expression in the gastrocnemius muscles, the same procedures were followed. Homogenates were centrifuged 10,000 g for 10 min at 4°C and analyzed for protein content (DC Protein Assay; Bio‐Rad Laboratories).

For analysis of phosphorylated and total p70S6K and 4EBP1 protein expression, working lysates were made at a concentration of 3 μg/μL in homogenizing buffer containing 4× Laemmli sample buffer (0.25 M Tris–HCl, pH 6.8, 6% SDS, 40% glycerol, 0.04% bromophenol blue, 16% DTT) and heated for 3 min at 95°C. Lysates containing 45 μg total protein were run on 4%–20% Criterion TGX Stain‐Free gels (Bio‐Rad Laboratories) and transferred to Immobilon‐P PVDF membrane (Merck Millipore, Burlington, MA, USA) for 1 h at 300 mA constant at room temperature (RT) in transfer buffer (41.2 mM Tris base, 173 mM glycine, 20% methanol). Membranes were blocked for 1 h at RT in 5% skim milk in Tris‐buffered saline‐Tween 20 (TBST) and incubated in primary antibodies diluted 1:1000 in 5% BSA/TBST overnight (o/n) at 4°C including rabbit‐α‐phospho‐p70 S6 kinase (RRID: AB_330944; T389; #9205 Cell Signaling Technology, Danvers, MA, USA) and rabbit‐α‐phospho‐4EBP1 (RRID: AB_330947; S65; #9451 Cell Signaling Technology). The following day, membranes were washed in TBST and incubated for 1 h at RT in Horseradish peroxidase (HRP)‐conjugated goat‐α‐rabbit IgG (H + L) (RRID: AB_11125142; #1706515; Bio‐Rad Laboratories) at 1:5000 in 5% BSA/TBST. After washing in TBST, membranes were incubated with enhanced chemiluminescence reagent (Immobilon Forte Western HRP substrate; Merck Millipore). For analysis of total p70S6K and 4EBP1 protein levels, membranes were stripped for 30 min in 0.25 M Tris–HCl, 2% SDS, 0.8% 2‐ME at 50°C, washed with TBST for 1–2 h at RT, blocked for 1 h at RT in 5% skim milk/TBST and incubated in primary antibodies diluted 1:1000 in 5% BSA/TBST o/n at 4°C including rabbit‐α‐p70 S6 kinase (RRID: AB_331676; #9202 Cell Signaling Technology) and rabbit‐α‐4EBP1 (RRID:AB_2097841; #9644 Cell Signaling Technology). The following day, membranes were washed in TBST and incubated for 1 h at RT in HRP‐conjugated goat‐α‐rabbit IgG (H + L) (RRID: AB_11125142; #1706515; Bio‐Rad Laboratories) at 1:5000 in 5% BSA/TBST. After washing in TBST, membranes were incubated with enhanced chemiluminescence reagent (Immobilon Forte Western HRP substrate; Merck Millipore).

For analysis of dystrophin and β‐dystroglycan protein expression, working lysates were made at a concentration of 2 μg/μL in homogenizing buffer containing 4× Laemmli sample buffer, and heated for 3 min at 95°C. Lysates containing 20 μg total protein were run on 4%–20% Criterion TGX Stain‐Free gels (Bio‐Rad Laboratories) and transferred to Immobilon‐P PVDF membrane (Merck Millipore) for 1 h at 300 mA constant at RT in transfer buffer (21.3 mM Tris base, 192 mM glycine, 10% methanol). Membranes were blocked for 1 h at RT in 5% BSA/TBST and incubated with primary antibodies diluted in 5% BSA/TBST o/n at 4°C, including mouse‐α‐dystrophin MANEX1011B(1C7) (RRID: AB_1157876; 1:1000; deposited by Morris, G.E; Developmental Studies Hybridoma Bank, The University of Iowa; USA) and mouse‐α‐β‐dystroglycan (RRID: AB_442043; 1:500; NCL‐b‐DG; Leica Biosystems). The following day, membranes were washed in TBST and incubated for 1 h at RT in HRP‐conjugated sheep‐α‐mouse IgG (RRID: AB_772210; #NA931; Cytiva Life Sciences, Sao Paulo, SP) at 1:5000 in 5% BSA/TBST. After washing in TBST, membranes were incubated with enhanced chemiluminescence reagent (Immobilon Forte Western HRP substrate; Merck Millipore). Samples were evaluated by integrated densitometry using a ChemiDoc XRS machine and Image Lab software (Bio‐Rad Laboratories). To determine the total protein signal for each sample, stainfree blots were imaged on the ChemiDoc XRS machine, according to manufacturer's instructions and evaluated by integrated densitometry using Image Lab software (Bio‐Rad Laboratories). For assessment of phosphorylated proteins, data were presented as the ratio of phosphorylated/total protein of interest. For assessment of non‐phosphorylated proteins, data were normalized to total protein based on densitometry of the stainfree blot. Data are presented relative to WT for all timepoints.

### 2.6 Statistical analyses

Data were analyzed between groups using either a one‐way or two‐way ANOVA as appropriate. When significance was detected with the ANOVA, a Tukey's post‐hoc multiple comparisons test was utilized to detect significant differences between means. A *p* value less than 0.05 was considered statistically significant. All statistical analyses were carried out using Prism GraphPad 6 software (GraphPad Software Inc., La Jolla, CA, USA). All values are presented as mean ± standard deviation (SD).

## 3 RESULTS

### 3.1 Denervation atrophy of the tibialis anterior, but not gastrocnemius or soleus muscles, is partially attenuated in DmdS3059E mice

**FIGURE 1:**
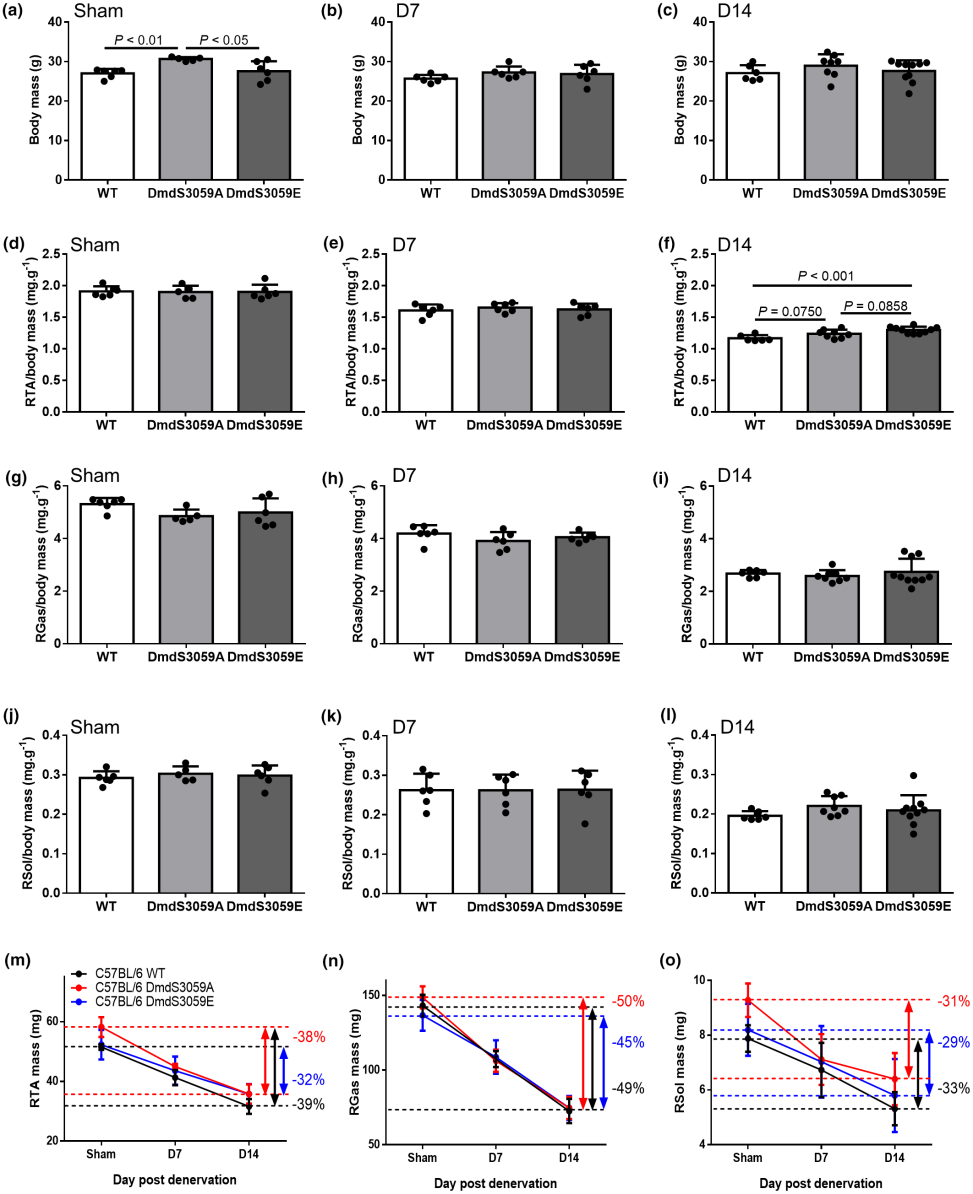
Mimicking phosphorylation of dystrophin S3059 partially attenuates wasting of mouse TA muscles after hindlimb denervation. Twelve‐week‐old male C57BL/6 WT, DmdS3059A, and DmdS3059E mice received either sham or denervation surgery and were then killed at 7 or 14 days later. Body mass was determined at endpoint in sham (a), D7 (b), and D14 (c) mice. Mass of the right tibialis anterior (RTA) muscle was assessed relative to body mass in sham (d), D7 (e), and D14 (f) mice. Mass of the right gastrocnemius (RGas) muscle was assessed relative to body mass in sham (g), D7 (h), and D14 (i) mice. Mass of the right soleus (RSol) muscle was assessed relative to body mass in sham (j), D7 (k), and D14 (l) mice. Absolute mass of the right TA (m), gastrocnemius (n), and soleus (o) muscles in sham mice and at D7 or D14 after denervation, depicting differences in the loss of absolute mass across the mouse strains. Data are presented as mean ± SD. *n* = 5–10. Statistical significances determined by a one‐way ANOVA followed by Tukey's multiple comparisons test.

To investigate the impact of dystrophin S3059 phosphorylation on denervation muscle atrophy, C57BL/6 WT, and C57BL/6 mice with a phospho‐null (DmdS3059A) or phospho‐mimetic (DmdS3059E) CRISPR‐mediated point mutation at S3059 of the dystrophin protein were subject to either a sham surgery (Sham) or sciatic nerve transection to induce denervation of the right hind limb and were euthanized for analysis either 7 or 14 days later. While DmdS3059A mice receiving the sham surgery were heavier than both WT (*p* < 0.01; Figure 1a) and DmdS3059E (*p* < 0.05; Figure 1a) mice, body mass was unchanged between genotypes in mice killed at 7 (Figure 1b) or 14 days (Figure 1c) after denervation. As expected, denervation induced atrophy of the tibialis anterior (TA; Figure 1d–f), gastrocnemius (Gas; Figure 1g–i), and soleus (Sol; Figure 1j–l) muscles relative to body mass at 7 and 14 days in all genotypes. Across genotypes, there was no difference in TA mass relative to body mass in mice receiving sham surgery (Figure 1d) or at 7 days after denervation (Figure 1e). However, at 14 days after denervation, TA mass relative to body mass was greater in Dmd3059E mice relative to WT mice (*p* < 0.001; Figure 1f), suggesting attenuated atrophy. TA mass relative to body mass at D14 in DmdS3059A mice was not significant relative to WT (*p* = 0.0750; Figure 1f) or DmdS3059E (*p* = 0.0868; Figure 1f) mice but intermediate between the two. This effect was specific to the TA muscle as the mass of both the gastrocnemius (Figure 1g–i) and soleus (Figure 1j–l) was unchanged between genotypes in mice receiving sham surgery and at 7 and 14 days after denervation. Attenuated TA muscle atrophy in DmdS3059E mice was confirmed by assessment of raw mass which showed a 32% reduction in DmdS3059E mice at D14 relative to sham, compared with 39% and 38% in WT and DmdS3059A mice, respectively (Figure 1m), which was less obvious in the gastrocnemius (Figure 1n) and soleus (Figure 1o) muscles.

### 3.2 TA muscles of DmdS3059A mice exhibit a shift towards a faster muscle phenotype

**FIGURE 2:**
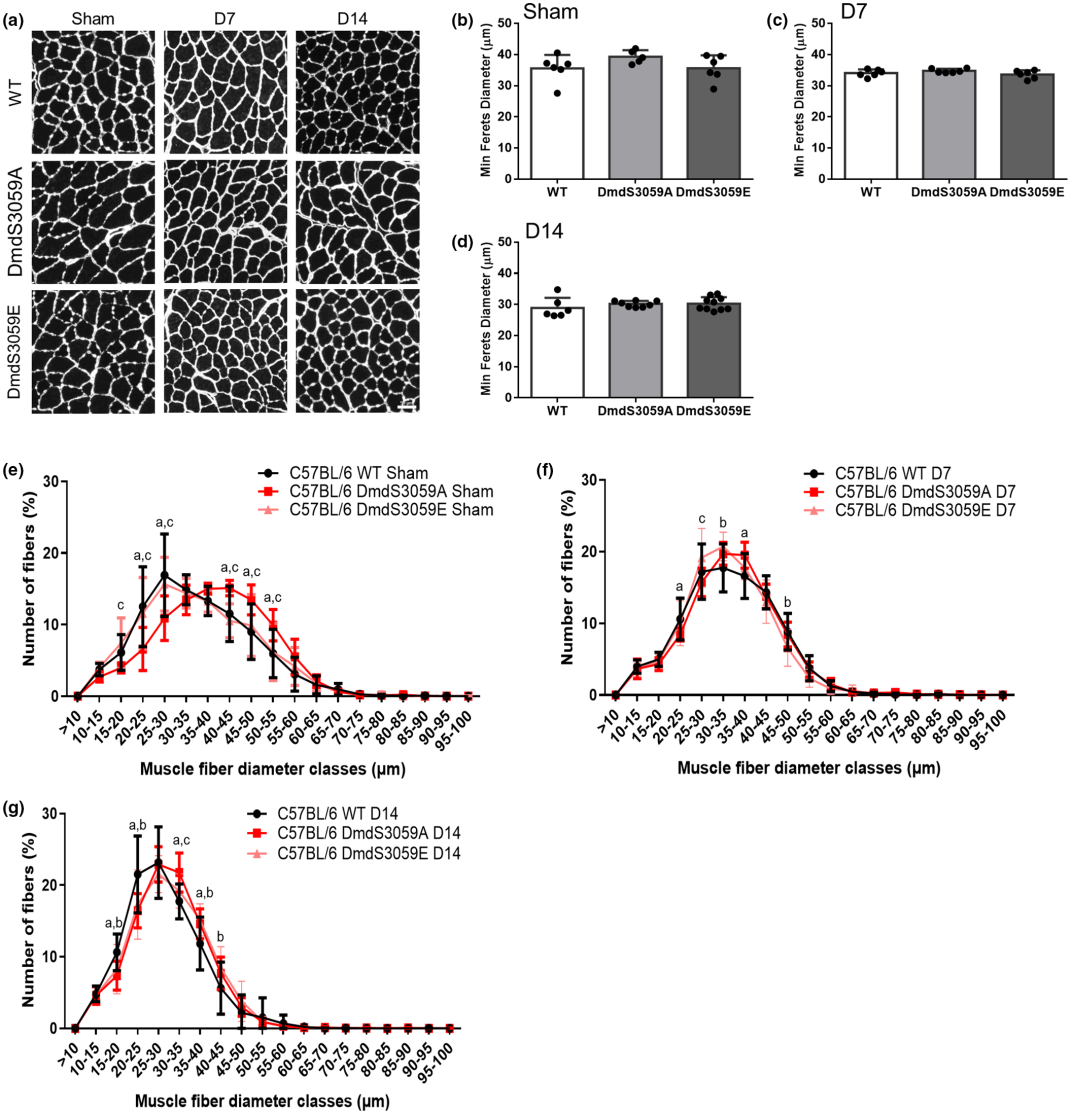
DmdS3059A mice have larger muscle fibers but atrophy to the same extent. Twelve‐week‐old male C57BL/6 WT, DmdS3059A, and DmdS3059E mice received either sham or denervation surgery and were killed 7 or 14 days after surgery. (A) Representative images of TA muscle sections from sham mice or mice taken at 7 or 14 days after denervation immunostained with antibodies to laminin. Scale bar = 50 μm. Average muscle fiber size was (minimum Feret's diameter) was determined in sham mice (B) and after 7 (C) or 14 (D) days of denervation. Data are mean ± SD. *n* = 5–10. Statistical significances determined using a one‐way ANOVA followed by Tukey's multiple comparisons test. Distribution of fiber size was expressed as a percentage of the total number of fibers in sham mice (E) and after 7 (F) or 14 (G) days post denervation. Data are represented as mean ± SD. *n* = 5–10. Statistical significance was determined by a repeated‐measures two‐way ANOVA followed by Tukey's multiple comparisons test, where (a) is significantly different between WT and DmdS3059A, (b) is significantly different between WT and DmdS3059E, and (c) is significantly different between DmdS3059A and DmdS3059E.

To further interrogate the changes in mass of sham and denervated TA muscles from WT, DmdS3059A, and DmdS3059E mice, fiber type composition, size, and oxidative capacity were examined. Average muscle fiber size (minimum Feret's diameter) was decreased from sham to D14 in all genotypes (Figure 2a‐d). Across the genotypes, there was no difference in average minimum Feret's diameter in mice receiving sham surgery (Figure 2a,b), or at 7 (Figure 2a,c) or 14 (Figure 2a,d) days after denervation. Minimum Feret's diameters were also binned to detect shifts in the proportion of fiber sizes. Such (potential) changes cannot be detected by assessing average minimum Feret's diameter alone. In mice receiving sham surgery there was a clear shift indicating the presence of larger muscle fibers in DmdS3059A mice with more muscle fibers with minimum Feret's diameters between 40 and 55 μm and fewer muscle fibers with minimum Feret's diameters between 15 and 30 μm, relative to WT and DmdS3059E mice (Figure 2e). At 7 days post denervation fiber proportions across the genotypes were similar, although minor changes persisted with fewer fibers having minimum Feret's diameters of 20–25 μm and more fibers with minimum Feret's diameters of 35–40 μm in DmdS3059A mice relative to WT; fewer fibers with minimum Feret's diameters of 30–35 μm but more fibers with minimum Feret's diameters of 45–50 μm in WT mice relative to DmdS3059E; and more fibers with minimum Feret's diameters of 25–30 μm in DmdS3059E mice relative to DmdS3059A (Figure 2f). Consistent with the observed changes in TA mass relative to body mass, by 14 days post denervation, the fiber proportions in DmdS3059A and DmdS3059E mice were similar and the distribution was shifted rightward, indicating larger muscle fibers, relative to WT mice (Figure 2g).

**FIGURE 3:**
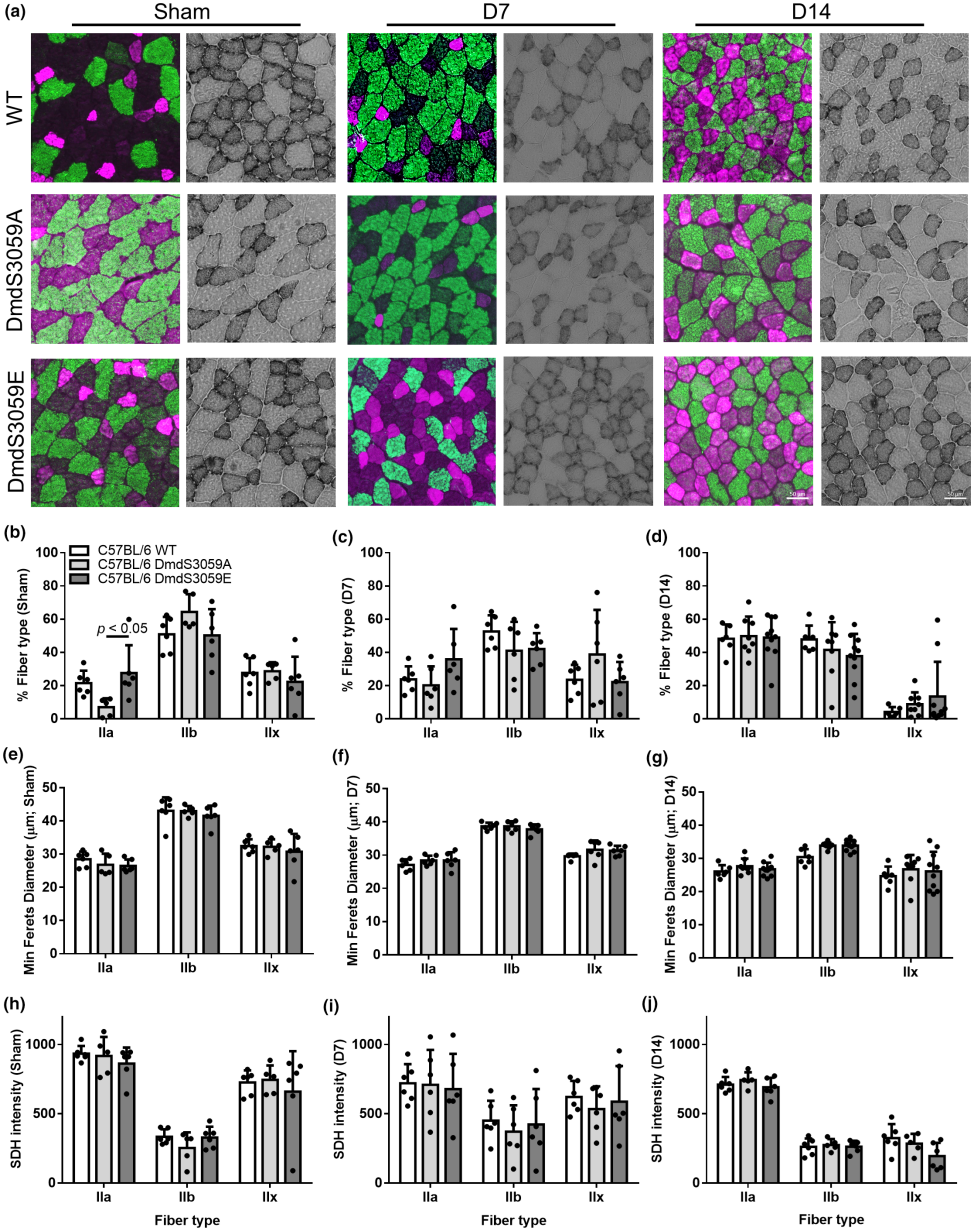
Fiber type analysis of TA muscles from C57BL/6 WT, DmdS3059A, and DmdS3059E mice at 7 and 14 days after denervation. Twelve‐week‐old male C57BL/6 WT, DmdS3059A, and DmdS3059E mice received either sham or denervation surgery and were then killed 7 or 14 days later. (a) Representative sections of TA muscles (Left panel) immunostained with antibodies to detect type IIa (purple) and type IIb (green) muscle fibers (unstained fibers (black) were assumed to be IIx); or (Right panel) reacted for SDH activity. Scale bar = 50 μm. The proportion of IIa, IIb, and IIx fibers was determined in sham mice (b), and at D7 (c) or D14 (d) after denervation. Size (minimum Feret's diameter) of IIa, IIb, and IIx muscle fibers was determined in sham mice (e), and at D7 (f) or D14 (g) after denervation. SDH intensity of the IIa, IIb, and IIx muscle fibers was determined in sham mice (h), and at D7 (i) or D14 (j) after denervation. Data are presented as mean ± SD. *n* = 5–10. Statistical significance determined by two‐way ANOVA followed by Tukey's multiple comparisons test.

Analysis of type IIa, IIb, and IIx fiber proportions in the TA muscle revealed all genotypes exhibited a similar shift towards a slow muscle phenotype by D14 post denervation, with an increase in the proportion of IIa muscle fibers and a concomitant decrease in IIx muscle fibers (Figure 3a–d). While no difference was observed in IIa, IIb, or IIx muscle fiber proportions between WT, DmdS3059A, or DmdS3059E mice at D7 (Figure3c) or D14 (Figure 3d) after denervation, there was a decrease in the proportion of IIa muscle fibers in the TA muscles from sham DmdS3059A mice relative to DmdS3059E mice (*p* < 0.05; Figure 3b) suggesting a shift towards a faster muscle phenotype in the TA muscles of DmdS3059A mice. While the average minimum Feret's diameter varied for denervated muscle fibers across the genotypes, IIb and IIx muscle fibers were smaller post‐denervation, but not different between genotypes (Figure 3e–g). Similarly, SDH activity decreased in IIa and IIx muscle fibers with denervation, but was not different between TA muscles of WT, DmdS3059A, or DmdS3059E mice (Figure 3h–j).

### 3.3 DGC protein expression increases in denervated muscles of DmdS3059E mice

**FIGURE 4:**
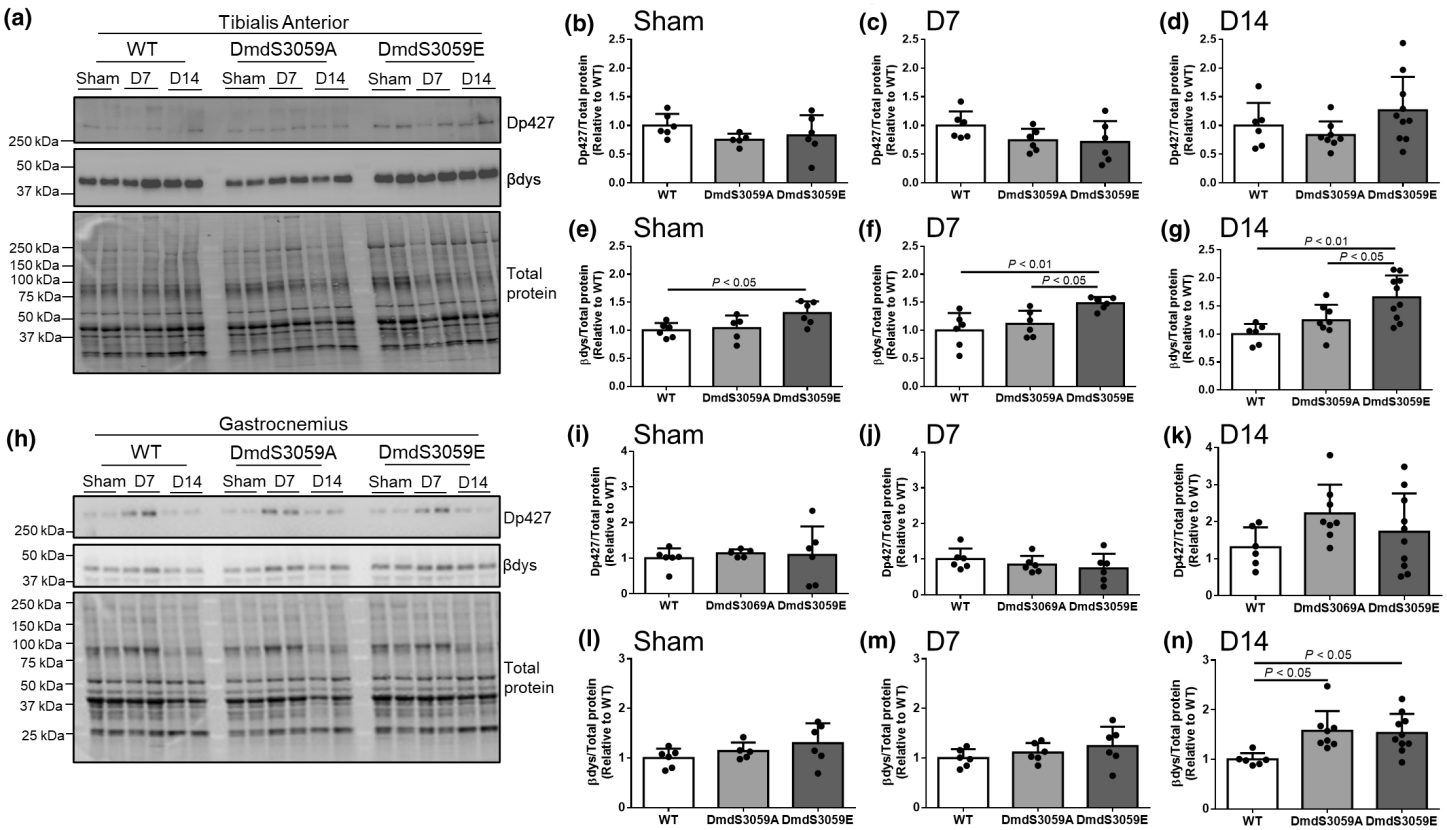
β‐dystroglycan expression is increased in TA muscles of DmdS3059E mice. Twelve‐week‐old male C57BL/6 WT, DmdS3059A, and DmdS3059E mice received either sham or denervation surgery and then killed at 7 or 14 days post denervation. (a) Muscles were lysed for protein extraction and analyzed by western immunoblotting for dystrophin (Dp427) and β‐dystroglycan. Expression of Dp427 and β‐dystroglycan was determined relative to total protein expression by densitometry of the stainfree blot, and expressed relative to WT, in sham (b, e), D7 (c, f), and D14 (d, g) mice. (h) Gastrocnemius muscles from sham mice or mice taken at 7 or 14 days post denervation were lysed for protein extraction and analyzed by western immunoblotting for dystrophin (Dp427) and β‐dystroglycan. Expression of Dp427 and β‐dystroglycan was determined relative to total protein expression by densitometry of the stainfree blot, and expressed relative to WT, in sham (i, l), D7 (j, m), and D14 (k, n) mice. Data are mean ± SD. *n* = 5–10. Statistical significance determined by one‐way ANOVA followed by a Tukey's multiple comparisons test.

As the difference between the mice was a single point mutation in the endogenous dystrophin protein, we examined the impact of these mutations on dystrophin and β‐dystroglycan protein expression in sham and denervated mice. In TA muscles, dystrophin protein expression was unchanged across genotypes in mice receiving sham surgery (Figure 4a,b) and at 7 days (Figure 4a,c) and 14 days (Figure 4a,d) after denervation, confirming that neither the DmdS3059A nor DmdS3059E altered endogenous dystrophin expression levels. However, β‐dystroglycan protein expression was higher in TA muscles from DmdS3059E mice relative to WT mice in both mice receiving sham surgery (Figure 4a,e; *p* < 0.05), and at 7 days (Figure 4a,f; *p* < 0.01) and 14 days (Figure 4a,g; *p* < 0.01) post denervation. Interestingly, β‐dystroglycan protein expression was also higher in TA muscles from DmdS3059E mice relative to DmdS3059A mice at 7 days (Figure 4a,f; *p* < 0.05) and 14 days (Figure 4a,g; *p* < 0.05) post denervation. In gastrocnemius muscles, where atrophy was not different across the genotypes, there was no change in dystrophin protein expression (Figure 4h–k). β‐dystroglycan protein expression was unchanged across genotypes in mice receiving sham surgery (Figure 4h,l), and at 7 days post denervation (Figure 4h,m). At 14 days post denervation, β‐dystroglycan expression was increased in both DmdS3059A and DmdS3059E mice relative to WT (Figure 4h,n; *p* < 0.05), but not different between DmdS3059A and DmdS3059E mice.

### 3.4 Changes in muscle mass at 14 days after denervation are unlikely to result from altered atrophy/growth signaling

**FIGURE 5:**
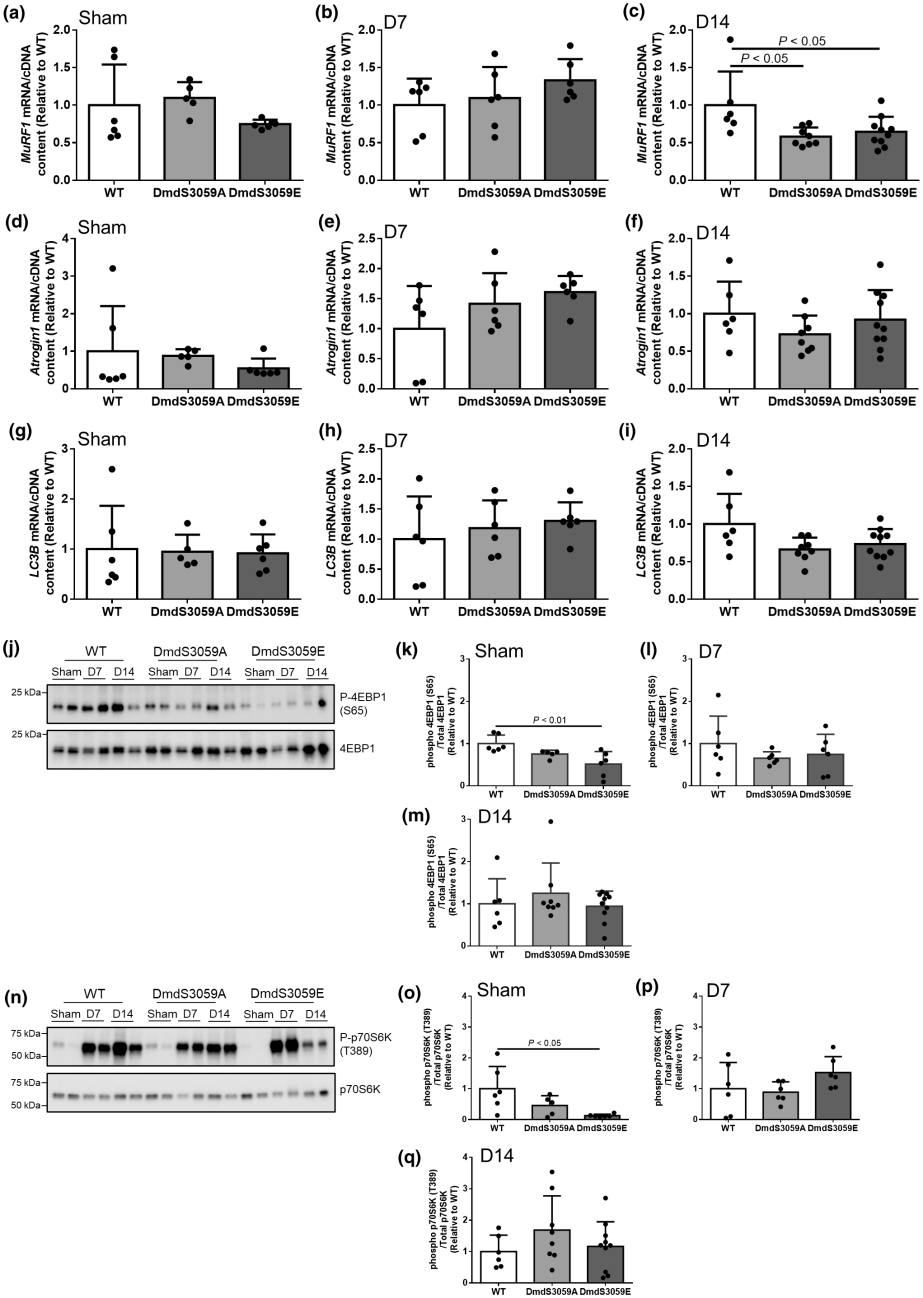
Protein synthesis and degradation pathways respond similarly after denervation in WT, DmdS3059A, and DmdS3059E mice. 12‐week‐old male C57BL/6 WT, DmdS3059A, and DmdS3059E mice received either sham or denervation surgery and were then killed 7 or 14 days later. RNA was extracted from half of the RTA muscle (that had been snap frozen in liquid N2), cDNA synthesized, and qRT‐PCR analysis undertaken for gene expression levels of *MuRF1*, *Atrogin1*, and *LC3B* normalized to total cDNA content, and expressed relative to WT, in sham (a, d, g), D7 (b, e, h), and D14 (c, f, i) mice. Lysate from right TA muscles analyzed by western immunoblotting for markers of protein synthesis. Expression of phosphorylated/total 4EBP1 (j) was quantified, and expressed relative to WT, in sham (k), D7 (l), and D14 (m) mice. Expression of phosphorylated p70S6K/total p70S6K (n) was quantified, and expressed relative to WT, in sham (o), D7 (p), and D14 (q) mice. Data are mean ± SD. *n* = 5–10. Statistical significance determined by one‐way ANOVA followed by a Tukey's multiple comparisons test.

Since muscle mass is tightly regulated by protein synthesis and protein degradation, we assessed whether increased TA mass in DmdS3059E mice relative to WT mice at 14 days after denervation was associated with altered muscle atrophy/growth signaling. There was no difference in *MuRF1* gene expression across the genotypes in mice receiving sham surgery (Figure 5a) or at D7 after denervation (Figure 5b). However, *MuRF1* gene expression was reduced in TA muscles from DmdS3059A and DmdS3059E mice relative to WT mice at D14 after denervation (*p* < 0.05; Figure 5c). Gene expression of *Atrogin1* (Figure 5d–f) and *LC3B* (Figure 5g–i) was unchanged between genotypes in mice receiving sham surgery and at D7 and D14 after denervation.

To assess changes in mTORC1‐mediated protein synthesis‐related signaling after denervation, we examined the phosphorylation status of the mTORC1 downstream signaling mediators, p70S6K and 4EBP1. Consistent with previous reports, p70S6K phosphorylation was increased at D7 and D14 after denervation across all genotypes (Figure 5j). When analyzed relative to WT mice, both 4EBP1 S65 (Figure 5j,k; *p* < 0.05) and p70S6K T389 phosphorylation (Figure 5n,o; *p* < 0.05) were decreased in DmdS3059E mice subjected to sham surgery. After denervation, there was no difference in 4EBP1 S65 phosphorylation in TA muscles at D7 (Figure 5j,l) or D14 (Figure 5j,m) across genotypes. Similarly, p70S6K T389 phosphorylation was not different across the genotypes at D7 (Figure 5n,p) or D14 (Figure 5n,q) after denervation, suggesting mTORC1 signaling was increased to similar levels across genotypes after denervation.

## 4 DISCUSSION

The multimeric DGC protein complex has a key function in maintaining striated muscle integrity during contraction via linkage of the extracellular matrix to the actin cytoskeleton (Ervasti & Campbell, 1993). Since the early 2000's, reports have suggested the DGC might also play a role in the regulation of skeletal muscle mass (Acharyya et al., 2005; Chockalingam et al., 2002; Hord et al., 2016; Kosek & Bamman, 2008). We, and others, have demonstrated that various post‐translational modifications to DGC protein members can alter the stability of the complex and that this might lead to alterations in muscle mass (Abdullah et al., 2020; Calderilla‐Barbosa et al., 2006; Fujimoto et al., 2020; Ilsley et al., 2001; James et al., 2000; Madhavan & Jarrett, 1994, 1999; Michalak et al., 1996; Miller et al., 2012; Milner et al., 1993; Ramirez et al., 2023; Senter et al., 1995; Shemanko et al., 1995; Sotgia et al., 2001; Swiderski et al., 2014, 2021; Wagner & Huganir, 1994; Zhou et al., 2006). We found that phosphorylation of S3059 within the cysteine‐rich region of the mouse dystrophin protein enhanced the binding affinity for dystrophin to β‐dystroglycan, which attenuated myotube atrophy in in vitro models of muscle wasting (Swiderski et al., 2014, 2021). Using mice with whole body CRISPR‐generated modifications of S3059 to either alanine to mimic a lack of phosphorylation, or glutamate to mimic phosphorylation on the dystrophin protein, we now show that phosphorylation of dystrophin S3059 can partially attenuate denervation atrophy in mouse TA muscles after sciatic nerve transection.

While our key observation was attenuated wasting of TA muscles in DmdS3059E mice at D14 relative to WT mice, it is worth noting that the DmdS3059A mutation resulted in an intermediate phenotype where TA mass was not significantly different from either WT or DmdS3059E mice. Consistent with this, while no difference was observed in average muscle fiber size across genotypes, both DmdS3059A and DmdS3059E mice had greater proportions of larger muscle fibers and smaller proportions of smaller muscle fibers relative to WT mice, at D14 post denervation. It is important to note that DmdS3059A mice had larger muscle fibers in the sham condition, suggesting that muscle fibers in these mice atrophied to a greater extent than those in DmdS3059E mice, highlighting a potential difference between the two strains. However, since phosphorylation is a dynamic process, likely to vary in response to a changing environment, it is perhaps unsurprising that changes might be observed in mice with constitutive changes to either a phospho‐null or phospho‐mimetic state relative to control. Interestingly, the expression of DGC proteins does vary with muscle fiber type, with higher expression in smaller, oxidative fibers (Omairi et al., 2019). Future studies examining changes in dystrophin S3059 phosphorylation state between specific fiber types may therefore be warranted to fully understand the role of this modification on dystrophin protein function.

Increased mTORC1 signaling after denervation has been considered to be a response to counteract atrophy (Quy et al., 2013), or to contribute to activation of the atrophy program (Tang et al., 2014). While mTORC1 signaling increased with denervation to a similar extent in all genotypes (indicated by increased phosphorylation of p70S6K at D7 and D14), it was interesting to observe decreased p70S6K and 4EBP1 phosphorylation at the basal state in TA muscles of DmdS3059E mice. Why this signaling pathway is reduced and specifically in the TA muscles of these mice, remains to be determined. More recently, skeletal muscle‐specific inducible knockdown of raptor (a key component of mTORC1) was used to demonstrate that protein synthesis is selectively upregulated in non‐type IIb muscle fibers after denervation to protect them from atrophy (You et al., 2021). While we did not assess activation of mTORC1 signaling in specific muscle fiber types in the present study, there were no differences in the size of IIa, IIb, or IIx muscle fibers between the genotypes in denervated TA muscles at D7 or D14. Therefore, the attenuated atrophy in TA muscles of DmdS3059E mice at D14 cannot be attributed to an increase in the size of a specific muscle fiber type. It is important to note that these studies were undertaken with mice in a fed state. Therefore, basal phosphorylation of these pathways is likely to be higher which may mask smaller changes induced by genotype or denervation. Further investigation in fasted mice may be warranted to interrogate this question in greater detail.

The key question arising from this study is why phosphorylation of dystrophin S3059 would partially attenuate atrophy in the TA but not in other hindlimb skeletal muscles. It is possible that alterations in muscle fiber composition could account for this effect, with an increasing proportion of type I fibers when comparing TA to gastrocnemius to soleus muscles (Augusto et al., 2004). Our assessment of DGC protein expression to try and understand the discrepancy between the muscles showed that although denervation increased β‐dystroglycan expression in the gastrocnemius at D14 in both DmdS3059A and DmdS3059E mice, β‐dystroglycan expression was increased only in DmdS3059E mice in the TA muscle of sham mice and at D7 and D14 post denervation. We, and others, have postulated the presence of a signaling pathway downstream of dystrophin/DGC that contributes to the regulation of skeletal muscle mass (Acharyya et al., 2005; Judge et al., 2006, 2011; Swiderski et al., 2021), but the existence of this pathway and its key players have yet to be identified. Early biochemical analyses of the DGC demonstrated associations with Ras and Rho GTPase signaling linked to tenotomy‐associated muscle atrophy (Chockalingam et al., 2002). As multiple GTPases, including RhoA, regulate mTORC1 signaling (Gordon et al., 2014), this could be one mechanism by which the DGC could regulate muscle mass. Such a mechanism has not been confirmed.

Lastly, since biochemical assessments of skeletal muscle protein synthesis and degradation provided no insight for why the TA muscles of DmdS3059E mice were larger than those from WT mice at D14 post denervation, it could be speculated that the increase in mass may be attributed to an accumulation of non‐muscle tissue. In the absence of timely reinnervation after denervation caused by tibial or sciatic nerve transection, fibrosis and/or fat can accumulate within skeletal muscles of rodents and humans (Borisov et al., 2001; Carlson, 2014; de Castro et al., 2007; Doherty et al., 2024; Dulor et al., 1998; Wagatsuma, 2006). Such changes can occur because of fibrogenic and adipogenic differentiation of fibro‐adipogenitor (FAP) cells (Doherty et al., 2024; Madaro et al., 2018). However, such fibro‐fatty changes are usually only observed as a long‐term consequence of denervation and generally not apparent until after the 15‐day timepoint in rodent models (Borisov et al., 2001; Doherty et al., 2024; Wagatsuma, 2006). While assessment of fibrosis and adipogenic tissue deposition was not assessed in this study, our histological assessments of muscle fiber type and size did not reveal significant non‐muscle tissue accumulation that required further investigation at D7 or D14 post denervation. Whether the small increase in TA mass in DmdS3059E mice at D14 is an early indication of accumulating non‐muscle tissue remains worthy of further investigation in longer‐term models.

In conclusion, mimicking constitutive phosphorylation of S3059 in the dystrophin protein conferred a small, but significant attenuation of (TA) muscle atrophy in mice after sciatic nerve transection. The level of attenuation was much smaller than that observed in our in vitro models of muscle wasting suggesting that the severity of denervation‐induced atrophy may be too great to be overcome by a single amino acid phosphorylation. S3059 phosphorylation may have greater protective benefits in other less severe in vivo models of muscle wasting.

## AUTHOR CONTRIBUTIONS

K.S., P.G., and G.S.L. designed the study. K.S., T.N., J.T., A.C., M.H., A.K., and C.A.G. performed the experimental work. K.S., P.G., and G.S.L. analyzed the data. K.S., C.A.G., P.G., and G.S.L. drafted the manuscript. All authors had the opportunity to comment on the manuscript. K.S. and G.S.L. edited and revised the manuscript for submission.

## FUNDING INFORMATION

This study was supported by the National Health and Medical Research Council of Australia (GRNT1144772). The author/s acknowledge the facilities, and the scientific and technical assistance of The Melbourne Advanced Genome Editing Centre (MAGEC). MAGEC is supported by Phenomics Australia (PA). PA is supported by the Australian Government through the National Collaborative Research Infrastructure Strategy (NCRIS) program.

## CONFLICT OF INTEREST STATEMENT

The authors declare that they have no conflict of interest.

## ETHICS STATEMENT

All experiments were approved by the Animal Ethics Committee of The University of Melbourne and conducted in accordance with the Australian code for the care and use of animals for scientific purposes as stipulated by the National Health and Medical Research Council (Australia).

## Supporting information


**Data S1:** Supporting Information.

## Data Availability

The authors confirm that the data supporting the findings of this study are available within the article [and/or] its supplementary materials.
