# Supplementary material for: Dystrophin S3059 phosphorylation partially attenuates denervation atrophy in mouse tibialis anterior muscles
Source: Physiol Rep. 2024 Jul 12;12(13):e16145. doi: 10.14814/phy2.16145 (PMC11245571; doi:10.14814/phy2.16145)
Supplement: Supplementary file 1 — Data S1: Supporting Information. [file PHY2-12-e16145-s001.pdf]

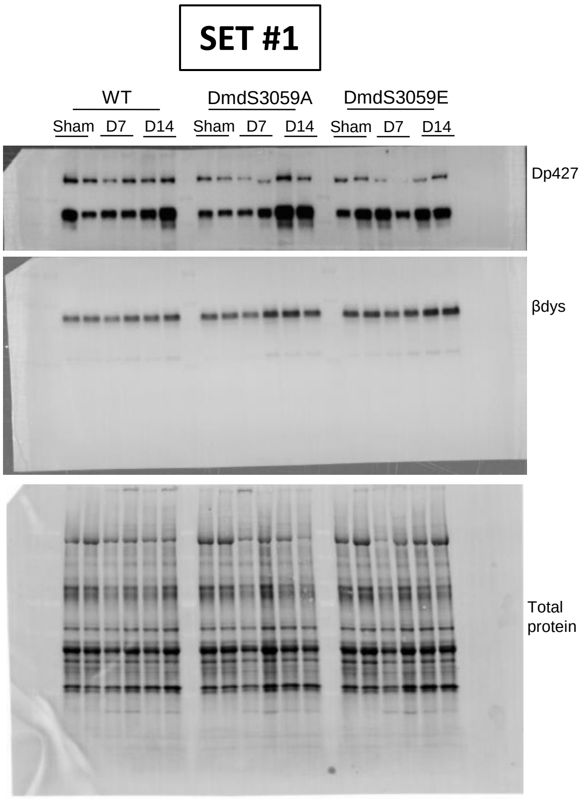

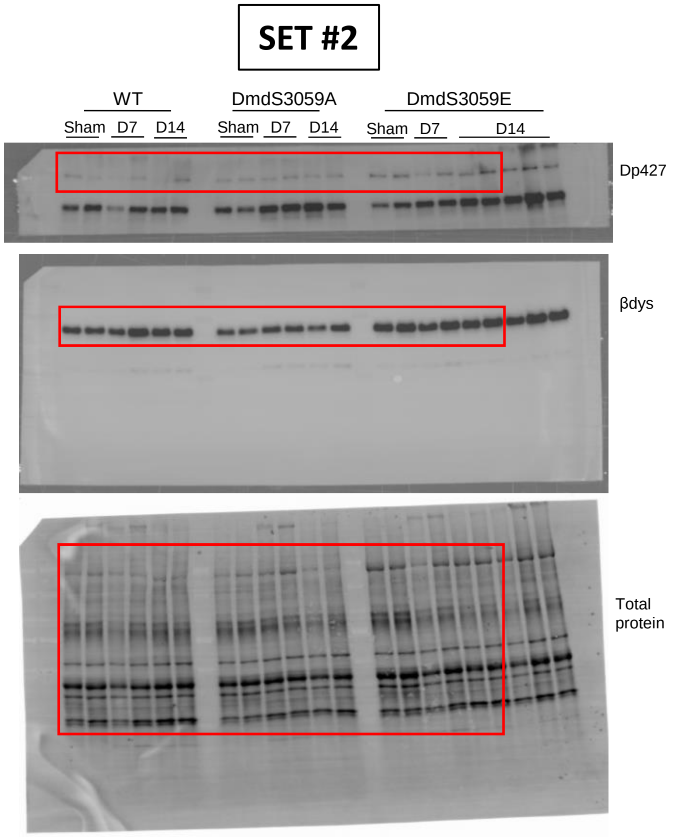

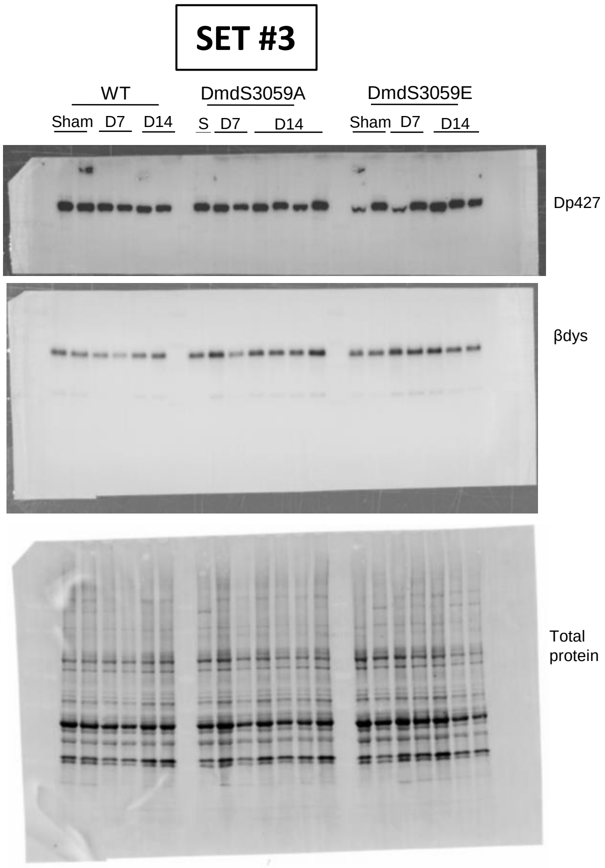

Fig. 4A Supp. Western blots

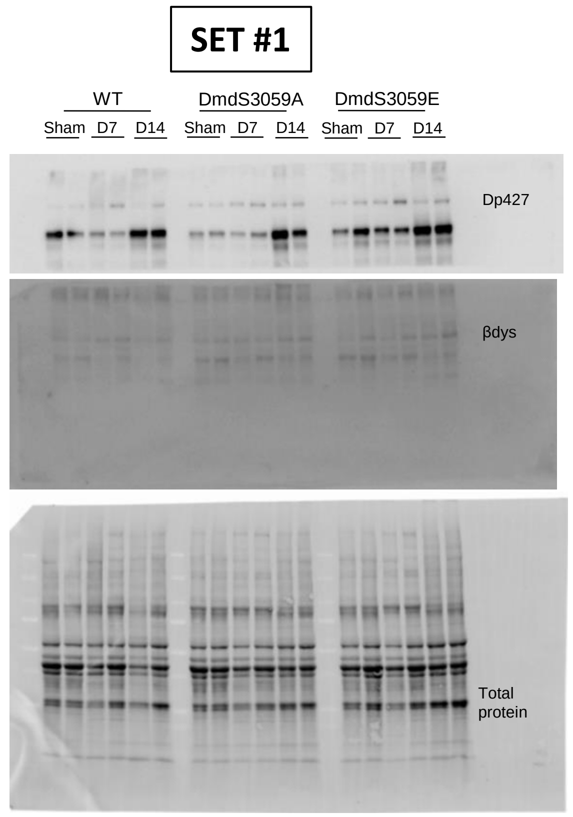

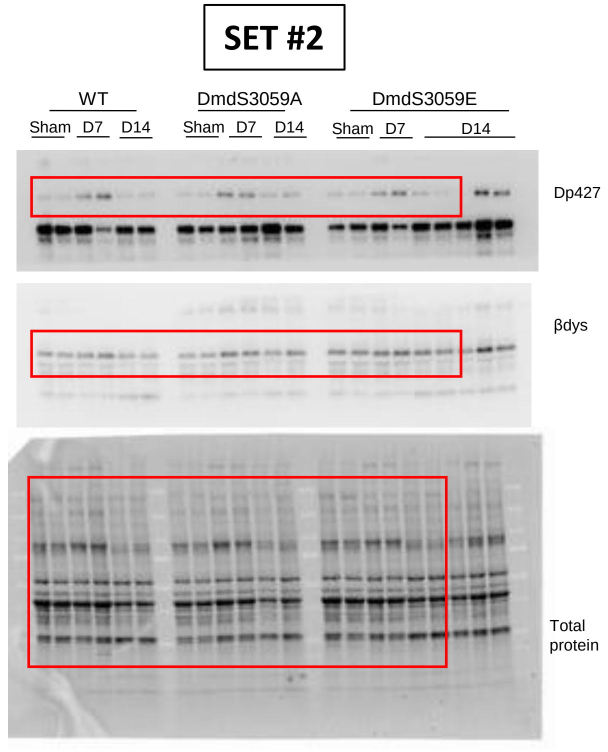

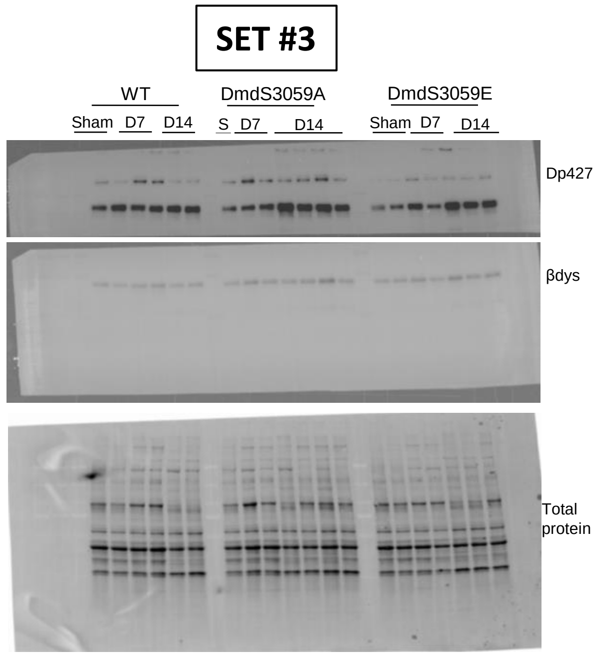

Fig. 4H Supp. Western blots

SET #1

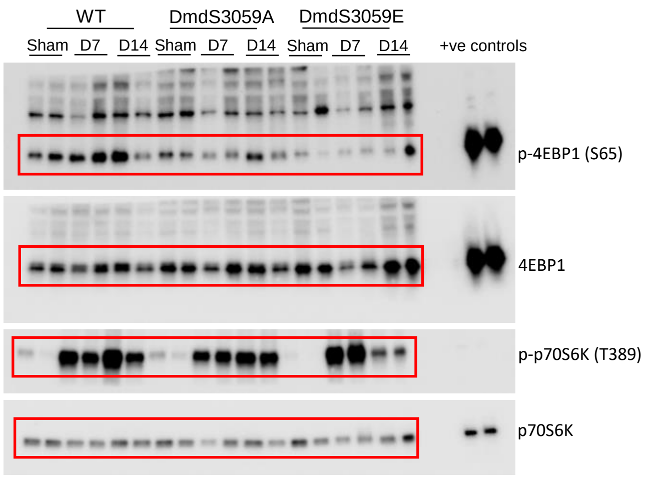

SET #2

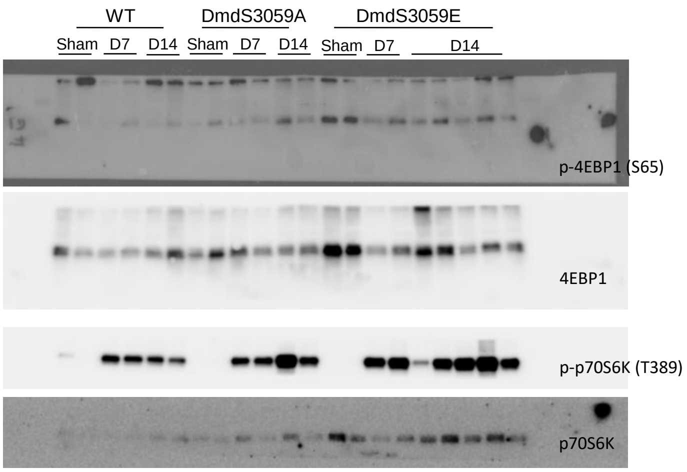

SET #3

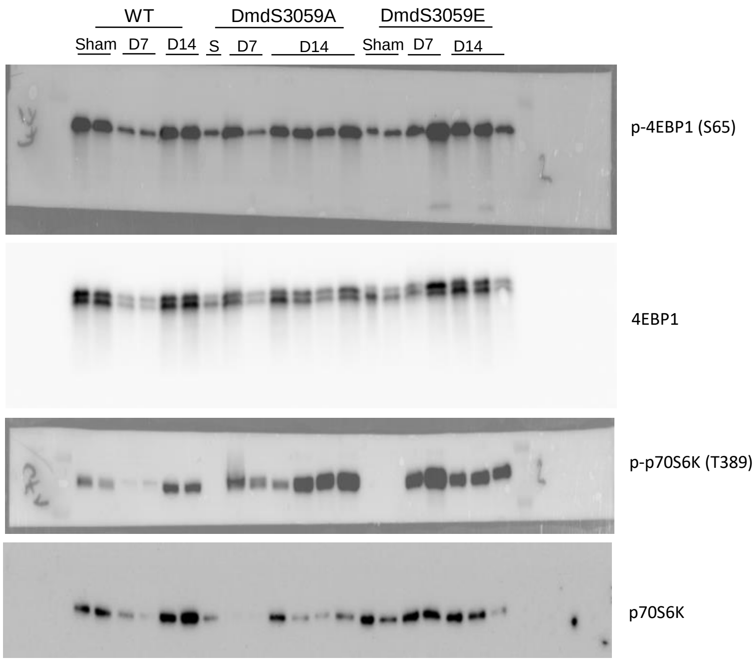

Fig. 5J+N Supp. Western blots
